# Thrombocytosis helps to stratify risk of colorectal cancer in patients referred on a 2-week-wait pathway

**DOI:** 10.1007/s00384-020-03597-9

**Published:** 2020-05-01

**Authors:** J. A. Bailey, N. Hanbali, K. Premji, J. Bunce, S. Mashlab, J. A. Simpson, D. J. Humes, A. Banerjea

**Affiliations:** 1 Nottingham Colorectal Service, Nottingham University Hospitals NHS Trust, E Floor West Block, QMC Campus, Nottingham, NG7 2UH UK; 2 Department of Clinical Chemistry, City Hospital, Nottingham University Hospitals NHS Trust, Nottingham, NG5 1PB UK; 3 Division of Epidemiology and Public Health, School of Medicine, University of Nottingham, Clinical Sciences Building, City Hospital, Nottingham, NG5 1PB UK

**Keywords:** Colorectal cancer, Thrombocytosis, Two-week-wait stratification, Risk stratification

## Abstract

**Purpose:**

Primary care studies suggest that thrombocytosis (platelet counts > 400 × 10^9^/L) is associated with an increased risk of colorectal cancer (CRC). We aimed to establish whether this marker has significant stratification value in patients seen in secondary care.

**Methods:**

A retrospective review of 2991 patients referred to our colorectal 2-week-wait (2WW) pathway between August 2014 and August 2017. Patient demographics were recorded prospectively, and local electronic records systems were used to retrieve full blood counts (FBC) and cancer diagnoses. Patients with no recent platelet count at the time of referral or incomplete records were excluded.

**Results:**

2236 patients were included in this evaluation. There was no significant difference in the age distribution of those with thrombocytosis and those without. There were significantly more females in the thrombocytosis group (72.1% vs 53.9%, chi-squared 24.63, *p* < 0.0001). 130 CRCs were detected (5.8%) and patients with thrombocytosis were more likely to have CRC (OR 2.62, 95% CI 1.60–4.30). The CRC diagnosis rate was significantly higher in females with thrombocytosis (10.3% vs 2.9%, chi-squared 19.41, *p* < 0.0001) and males with thrombocytosis (16.1% vs 7.9%, chi-squared 4.62, *p* = 0.032).

**Conclusion:**

Thrombocytosis appears to have stratification value in the 2WW population. Further evaluation of its value alone or in combination with other stratification tests is required.

## Introduction

Colorectal cancer (CRC) is common with around 42,000 new diagnoses made annually in the UK [1]. Outcomes in the UK lag behind the rest of Europe despite nearly two decades of 2-week-wait (2WW) pathways and other targets introduced to address this issue [2]. The desire for diagnosis at an earlier stage led to the introduction of broader referral criteria for CRC in 2015 with the aim of investigating all those with a risk of CRC ≥ 3%. However, these criteria for urgent referral to secondary care are largely based on patient age and symptoms [3]—the latter are often associated with later-stage disease and are inherently non-specific. The search for objective markers that may help to stratify risk remains attractive in this context.

We introduced straight to test (STT) colonoscopy in 2014 as part of our 2WW pathway [4]. A full blood count (FBC) was specified for inclusion with every referral. We have previously demonstrated the value of anaemia in those patients referred on an urgent pathway although compliance with submission of FBC results has been poor [5, 6]. An FBC also provides a platelet count and thrombocytosis (platelet count > 400 × 109/L) appears to have value for risk stratification of colorectal cancer in primary care [7]. We aimed to evaluate its utility in the secondary care setting by undertaking a review of existing data for patients referred on a 2WW colorectal cancer pathway at our institution. We report on its value as a single marker of risk, as well as its association with other recognised parameters such as age, sex and anaemia.

## Methods

### Data sources

Adult patients referred to the Colorectal Service at Nottingham University Hospitals under the 2WW pathway for CRC are prospectively recorded on a local database in accordance with best practice guidance for audit of straight to test pathways. The name, date of birth, age, sex, hospital ID, NHS number, date of referral and indication for referral is recorded for each patient.

Data for haemoglobin (Hb) and platelet counts at the time of referral were collected from the hospital electronic reporting system retrospectively. Cancer diagnoses, CRC and other cancer (OC) outcomes were collected from hospital electronic reporting system.

### Cohort

All patients referred under the 2WW pathway between 01 August 2014 and 31 August 2017 for suspected CRC were identified from the referral database populated by specialist nurses at the Colorectal Service at Nottingham University Hospitals NHS Trust. Duplicate and rejected referrals were identified and excluded. Patients with no full blood count (FBC) on referral, no investigations or unknown outcome were excluded from subsequent analysis of outcomes.

### Exposure and covariates

Anaemia was diagnosed according to the World Health Organization (WHO) definitions of a haemoglobin of < 120 g/L in women or < 130 g/L in men, based on the most recent Hb at the time of referral. Thrombocytosis was defined as platelets > 400 × 10^9^/L in line with primary care studies [7]. The presence or absence of anaemia and thrombocytosis was evaluated for all patients.

### Outcome definition

Colorectal cancer diagnosis was determined from investigation outcomes. Evidence of lower GI malignancy on colonoscopy, CT scans and histology reports reviewed at our cancer multi-disciplinary team (MDT) meeting that confirmed adenocarcinomas were reviewed for diagnosis. Non-colorectal cancer diagnoses were also recorded.

### Statistical analysis

Data were assessed for normality using histograms and a Shapiro-Wilk test. Comparisons were made between continuous variables using the Student *t* test if normally distributed or Mann-Whitney if not normally distributed. Categorical data was summarised using frequencies and percentages. Missing data were classified in a separate category and included in models. Comparisons were made between categorical data using chi-squared tests. Logistic regression analysis was used to test the association between diagnosis of colorectal cancer and thrombocytosis accounting for age, gender and anaemia. Univariate analysis was undertaken with age as a continuous variable; a multivariate model was then built including all factors associated with colorectal cancer in the univariate analysis. All statistics were performed using Stata Version 16 (Stata Corp, College Station, TX, USA). Tests of significance were considered significant if a *p* value of less than 0.05 was obtained.

## Results

A total of 2991 patients referred for 2WW investigation during the study period were available for review; 2236 (74.8%) were included in the analysis. 755 patients (25.2%) were excluded from the study. In total 394 (13.2%), had no FBC available, 225 (7.5%) were not investigated due to clinical judgement/patient choice, 82 (2.7%) had missing clinic/blood test information, 45 (1.5%) did not attend their appointment and 9 (0.3%) were excluded due to database error/duplication.

Demographic characteristics are shown in Table 1. There was no significant difference in the age distribution of those with thrombocytosis and those without. 55.5% of the cohort were females; there were significantly more females in the thrombocytosis group than the group with normal platelets (72.1% vs 53.9%, chi-squared 24.63, *p* < 0.0001).

**Table 1:** The proportion of patients diagnosed with CRC; univariate and multivariate analysis for thrombocytosis accounting for age, gender and anaemia. Patients with thrombocytosis were more likely to have CRC (OR 2.62, 95% CI 1.60–4.30, *p* = < 0.001) but not non-colorectal cancers (analysis not shown—OR 0.76, 95% CI 0.26–2.18)

Parameter	Total	CRC	Univariate analysis		Multivariate analysis	
Total patients	2236	130	OR (95% CI)	*p* value	OR (95% CI)	*p* value
Age (SEM)	68.19 (0.27)	74.22 (0.87)	1.05 (1.03–1.07)	< 0.001	1.04 (1.02–1.06)	< 0.001
Male (%):female (%)	994 (44.5%):1242 (55.5%)	83 (63.8%):47 (36.2%)	2.32 (1.60–3.35)	< 0.001	2.44 (1.67–3.58)	< 0.001
Anaemia (%)	860 (38.5%)	82 (63.1%)	2.74 (1.92–3.93)	< 0.001	1.83 (1.23–2.73)	0.003
Thrombocytosis (%)	201 (9.0%)	25 (19.2%)	2.51 (1.58–3.98)	< 0.001	2.62 (1.60–4.30)	< 0.001

*SEM* standard error of mean

A total of 130 CRCs (5.8%) and 52 other cancers (2.3%) were diagnosed in this cohort. CRC diagnosis was more likely in patients with thrombocytosis (12.4% vs 5.2%, chi-squared 17.70, *p* = < 0.0001) compared with those with a normal platelet count, significant for both females (11% vs 2.8%, chi-squared 23.70, *p* = < 0.0001) and males (16.1% vs 7.9%, chi-squared 4.62, *p* = 0.032). Thrombocytosis was significantly associated with advanced (stage 3/4) CRC diagnosis (19.1% vs 8.5%, chi-squared 14.4, *p* < 0.0001) and with a diagnosis of right-sided cancers (34% vs 9.6%, chi-squared 11.87, *p* = 0.001) vs left-sided and rectal cancers. Patients with anaemia were also significantly more likely to be diagnosed with CRC (9.5% vs 3.5%, chi-squared 35.33, *p* < 0.0001) compared with those with a normal haemoglobin.

Univariate analysis identified sex, age, anaemia and thrombocytosis as significant risk factors for CRC diagnosis as summarised in Table 1. Multivariate logistic regression of the whole dataset confirmed the association of thrombocytosis with CRC diagnosis after adjustment for gender, age and anaemia (OR 2.62, 95% CI 1.60–4.30). Repeat analysis was completed with non-CRCs excluded, yielding the same results (OR 2.58, 95% CI 1.57–4.25). No association was found in a comparative analysis on thrombocytosis for the diagnosis of non-CRCs (OR 0.76, 95% CI 0.26–2.18).

## Discussion

We demonstrate that thrombocytosis has value in stratifying risk for this cohort of patients after referral on a 2WW pathway for CRC. Our study period mostly pre-dates the Bailey et al. paper [8] highlighting the value of platelets in primary care risk stratification, and it is unlikely that this publication would have affected our results. We note that thrombocytosis appears more significant in our female population—the reverse of the primary care study in which males showed a greater increase in risk. However, symptoms such as change in bowel habit are more common in females than males, leading to more of this group being referred on a 2WW pathway despite a lower risk of CRC. This population is therefore selected from the general population on the basis of symptoms, which may also explain the lack of increased risk for other malignancies. As the risk of CRC increases with age, it is understandable that the CRC group selected from a 2WW cohort is comparatively older. In our dataset, there was no significant association between age and thrombocytosis. Overall, we demonstrate that thrombocytosis confers an increased CRC risk in the referred population independent of gender, age and anaemia.

An FBC is a cheap easily accessible test that appears to provide two objective markers of risk in haemoglobin and platelet count [5, 8]. However, despite our referral form requesting submission of this data since 2014, only 82.8% of referrals complied with this. As a retrospective study of thrombocytosis, there was little choice but to exclude those without a recorded FBC (394), which is a limitation of our study. Local discussions with primary care commissioners identified a concern that waiting for test results to make a referral may cause delays in suspected cancer cases. The 2WW initiative in the UK requires that a GP suspecting a diagnosis of cancer sends a referral to the secondary care provider within 24 h, without pre-requisite tests stipulated [9]. Following integration of the Faecal Immunochemical Test (FIT) into our urgent pathway in 2019, an FBC is now mandatory at the time of referral. Incomplete referrals are returned for completion.

Within this cohort, 5.8% of patients were diagnosed with CRC, satisfying the 3% NICE threshold for urgent investigation. However, the 2.9% detection rate in women without thrombocytosis suggests some subsets of this cohort may fall below that cut-off. The presence of thrombocytosis in 19.2% of CRC diagnoses reaffirms the notion that a single parameter is insufficient for risk stratification.

Association between thrombocytosis and right-sided cancers is of great interest, given the increased likelihood of false-negative FIT results from right-sided cancers [10]. In our cohort, thrombocytosis was also significantly associated with later-stage CRC, though not specifically metastatic cancer. In some cases within this dataset, thrombocytosis was the only abnormal stratification parameter in early-stage CRC.

Identifying cohorts at increased risk of CRC is key to improving diagnosis at earlier stages when outcomes are favourable [2]. Increasing demand for 2WW diagnostic capacity requires more individualised risk stratification in order to improve rates of early diagnosis and clinical effectiveness of such pathways [11]. We have combined anaemia with faecal immunochemical testing (FIT) in our local pathway [12], and whilst FIT is useful at the extremes of faecal Hb (f-Hb) concentrations, there is a broad range (locally between 4 and 100) that would benefit from additional discriminatory value. Scoring systems such as FAST hold promise in this regard [13]. Here, we postulate that adding values from an FBC such as Hb and platelet count may improve the performance characteristics of such a score. It might be argued that such parameters could be extrapolated to support individualised decision-making in screening programmes to further decrease CRC mortality and minimise iatrogenic harm [14]. The English Bowel Cancer Screening Programme could recommend an FBC to identify anaemia or thrombocytosis meriting further investigation for patients with faecal haemoglobin concentrations below the 120-μg Hb/g faeces cut-off threshold.
